# Peripheral Brain-Derived Neurotrophic Factor Changes and Clinical Response to Electroconvulsive Therapy and Repetitive Transcranial Magnetic Stimulation in Major Depressive Disorder: A Systematic Review

**DOI:** 10.7759/cureus.119286

**Published:** 2026-11-07

**Authors:** Christian David Galindo, Guillermo Jemar, Isabel Carolina Osorio, Andres Lopez, Mariana Uribe Zapata, Laura Gonzalez Perez, Sebastian D Villamil Montejo, Luis Antonio Carvajal Angarita

**Affiliations:** 1 Psychiatry, Universidad internacional Valencia, Valencia, ESP; 2 Psychiatry, Corporación Universitaria Remington, Medellín, COL; 3 Psychiatry and Behavioral Sciences, Hospital Mental de Antioquia María Upegui, Medellin, COL; 4 Psychiatry, Hospital Interdisciplinario Psicoasistencial José Tiburcio Borda, Buenos Aires, ARG; 5 Psychiatry and Forensic Medicine, Universidad de Buenos Aires, Buenos Aires, ARG; 6 Psychiatry and Behavioral Sciences, Universidad Maimónides, Buenos Aires, ARG; 7 Psychiatry, E.S.E. (Empresa Social del Estado) Hospital Carisma, Medellín, COL; 8 Child and Adolescent Psychiatry, Universidad El Bosque, Bogotá, COL; 9 Psychiatry, Clinica Samein, Medellín, COL; 10 Psychiatry and Behavioral Sciences, EIA University, Medellín, COL; 11 College of Medicine, Universidad Nacional De Colombia, Bogotá, COL; 12 Psychiatry and Behavioral Sciences, Corporación Universitaria Remington, Medellín, COL

**Keywords:** brain-derived neurotrophic factor (bdnf), electroconvulsive therapy (ect), major depressive disorder, neurogenesis and neuroplasticity, repetitive transcranial magnetic stimulation (rtms), systematic literature review

## Abstract

Major depressive disorder (MDD) is a leading contributor to the global burden of disease, and a substantial proportion of patients experience inadequate responses to conventional treatments. Brain-derived neurotrophic factor (BDNF), a key regulator of neuronal survival and synaptic plasticity, has been investigated as a potential biomarker of antidepressant response. However, its clinical relevance following brain stimulation remains uncertain. This systematic review evaluated changes in peripheral BDNF concentrations and their association with clinical outcomes following electroconvulsive therapy (ECT) and repetitive transcranial magnetic stimulation (rTMS) in adults with MDD. The review followed Preferred Reporting Items for Systematic reviews and Meta-Analyses (PRISMA) 2020 guidelines, and searches were conducted in PubMed/MEDLINE (Medical Literature Analysis and Retrieval System Online), PsycINFO, and LILACS (Latin America and the Caribbean Literature on Health Sciences). Study selection and data extraction were independently performed by two reviewers, with disagreements resolved through consensus or consultation with a third reviewer. Eleven studies met the eligibility criteria, including two randomized controlled trials and nine non-randomized investigations. Due to substantial clinical and methodological heterogeneity, a meta-analysis was not performed, and findings were synthesized narratively. Peripheral BDNF responses varied across interventions, biological matrices, and sampling intervals. While many studies reported increased serum or plasma BDNF following ECT, others documented substantial clinical improvement without corresponding biomarker changes. Evidence following rTMS was less consistent, with insufficient data to establish a reliable association between BDNF changes and antidepressant response. Overall, peripheral BDNF changes did not consistently parallel clinical improvement, and current evidence does not support their use as a standalone biomarker of antidepressant response. Further adequately powered studies with standardized biomarker measurements and longitudinal clinical assessments are warranted.

## Introduction and background

Major depressive disorder (MDD) is a prevalent, recurrent, and disabling psychiatric disorder that contributes substantially to the global burden of disease [1]. Beyond its effects on quality of life, psychosocial functioning, and occupational performance, MDD is associated with increased medical comorbidity, premature mortality, and considerable socioeconomic costs [2]. Despite advances in pharmacological and psychotherapeutic interventions, findings from the Sequenced Treatment Alternatives to Relieve Depression (STAR*D) trial indicated that approximately one-third of patients achieved remission following an initial antidepressant treatment step, highlighting the limitations of first-line treatment [3]. Furthermore, a substantial proportion of patients experience persistent depressive symptoms despite multiple adequately administered interventions, underscoring the clinical challenge of treatment-resistant depression (TRD) [4]. These therapeutic limitations have intensified efforts to identify biological mechanisms and clinically informative biomarkers that could improve treatment selection, facilitate therapeutic monitoring, and support the development of precision psychiatry approaches [5].

Brain stimulation therapies have become important treatment options for patients with severe or treatment-resistant depressive episodes [6]. Electroconvulsive therapy (ECT) remains among the most effective interventions for severe depression, particularly when rapid clinical improvement is required or when psychotic symptoms, catatonia, or substantial suicide risk are present. Repetitive transcranial magnetic stimulation (rTMS) provides a non-invasive alternative with demonstrated antidepressant efficacy, while other techniques, including transcranial direct current stimulation (tDCS), have also been investigated as therapeutic options [7]. Advances in stimulation protocols, including theta burst stimulation and accelerated approaches, have further expanded the field of therapeutic neuromodulation. Nevertheless, the neurobiological mechanisms underlying antidepressant response to these interventions remain incompletely understood, and the identification of reliable biomarkers continues to represent an important research priority [8].

Alterations in neuroplasticity have been implicated in the pathophysiology of MDD and may contribute to the biological effects of antidepressant treatments. Brain-derived neurotrophic factor (BDNF), one of the most extensively investigated neurotrophins in affective neuroscience, regulates neuronal survival, dendritic remodeling, synaptic plasticity, and activity-dependent neuronal adaptation [9]. These functions are particularly relevant to corticolimbic networks involved in emotional regulation, cognition, and stress responsiveness. Preclinical investigations have demonstrated that chronic stress can alter BDNF expression and signaling in brain regions implicated in depression, including the hippocampus and prefrontal cortex, whereas antidepressant interventions may modify neurotrophic pathways and promote structural and functional adaptations [10]. These observations have contributed to the neurotrophic hypothesis of depression, although the relationship between BDNF signaling and antidepressant response is complex and cannot be explained by changes in a single biological pathway.

Clinical investigations have frequently reported lower peripheral BDNF concentrations in patients experiencing major depressive episodes compared with healthy individuals. However, associations between circulating BDNF, depressive symptom severity, and treatment outcomes have been inconsistent across studies [11]. Serum and plasma BDNF measurements offer a relatively accessible approach to investigating treatment-associated biological changes, but their interpretation requires caution. Circulating concentrations are influenced by peripheral sources, platelet-related processes, and preanalytical conditions, and therefore cannot be considered direct indicators of central nervous system neuroplasticity. Consequently, although peripheral BDNF remains a biologically plausible candidate biomarker, its clinical validity requires evidence that measured concentrations provide reliable information about antidepressant outcomes beyond established clinical assessments.

The potential involvement of neurotrophic signaling in the antidepressant effects of brain stimulation has prompted investigations of peripheral BDNF concentrations before and after treatment [12]. Experimental studies suggest that electroconvulsive stimulation may influence hippocampal neurogenesis, synaptic remodeling, and BDNF-related signaling, whereas rTMS may modulate activity-dependent plasticity through changes in cortical excitability and neuronal network activity. These mechanisms provide a biological rationale for examining whether serum or plasma BDNF concentrations change following neuromodulation and whether such changes correspond to improvements in depressive symptoms [13]. Nevertheless, clinical findings remain inconsistent. Some investigations have documented increased peripheral BDNF following treatment, whereas others have reported substantial reductions in depressive symptom severity without significant changes in circulating concentrations [14]. Importantly, treatment-associated biomarker changes, correlations with symptom improvement, and the ability to predict clinical response represent distinct outcomes that should not be interpreted interchangeably.

Interpretation of these findings is complicated by methodological and biological heterogeneity. Differences in stimulation modality and parameters, baseline depression severity, treatment resistance, concomitant pharmacotherapy, biological sampling intervals, laboratory assays, and clinical outcome definitions limit comparability across investigations. Furthermore, serum and plasma BDNF concentrations are not directly interchangeable, and variation in sample processing may contribute to inconsistent results independently of treatment effects. These limitations are particularly relevant when evaluating whether peripheral BDNF can distinguish clinical responders from nonresponders or provide meaningful information about remission. Accordingly, the relationship between peripheral neurotrophic changes and antidepressant outcomes following brain stimulation remains insufficiently established [15].

Previous systematic reviews and meta-analyses have examined BDNF changes following specific brain stimulation modalities, contributing important evidence regarding treatment-associated neurotrophic responses [16]. However, uncertainty persists regarding the consistency of these changes across ECT and rTMS and, more importantly, their association with clinically relevant outcomes such as reductions in depressive symptom severity, treatment response, and remission. Distinguishing changes in circulating BDNF from their potential clinical significance is essential for evaluating whether peripheral measurements offer information beyond established symptom-based assessments. An integrated synthesis emphasizing these relationships may help clarify the current evidentiary limitations and identify methodological priorities for future biomarker research [17].

Accumulating evidence suggests that the relationship between peripheral BDNF dynamics and the antidepressant effects of brain stimulation therapies is more complex than initially hypothesized. Although BDNF has been extensively investigated as a candidate biomarker of neuroplasticity in MDD, its clinical validity as an indicator of antidepressant response remains to be established. Experimental and clinical investigations support the involvement of BDNF in synaptic plasticity, neuronal survival, neurogenesis, and activity-dependent neuronal remodeling. Nevertheless, the biological plausibility of these mechanisms does not necessarily establish that circulating BDNF concentrations accurately reflect treatment-induced neuroplastic changes within the central nervous system. This distinction is essential when considering peripheral BDNF as a potential biomarker of therapeutic efficacy [18].

Across brain stimulation modalities, the available literature reveals substantial variability in peripheral BDNF responses. ECT has frequently been associated with increased circulating BDNF concentrations following treatment; however, these biological changes do not consistently parallel improvements in depressive symptoms. Similarly, investigations involving rTMS have yielded inconsistent findings, with some reporting changes in neurotrophic concentrations and others documenting clinical improvement without corresponding alterations in peripheral BDNF. These observations indicate that changes in circulating BDNF do not consistently characterize antidepressant response across neuromodulation modalities, highlighting the complexity of the relationship between neurotrophic signaling and clinical outcomes [19].

The variability observed across studies may be partly explained by differences in clinical characteristics and methodological approaches. Patient selection, baseline depression severity, treatment resistance, stimulation parameters, treatment duration, concomitant pharmacotherapy, and definitions of clinical response may influence the interpretation and comparability of reported findings. Furthermore, differences in biological specimen type, blood collection timing, sample processing, and laboratory quantification methods introduce additional sources of variability. Serum and plasma BDNF measurements are particularly susceptible to differences in platelet-related release and preanalytical conditions, limiting their direct comparability. Physiological and environmental factors, including age, sex, physical activity, metabolic status, and inflammatory processes, may further affect circulating BDNF concentrations independently of antidepressant response [20].

Beyond methodological considerations, the biological interpretation of peripheral BDNF measurements remains a fundamental challenge. Although neurotrophic signaling is implicated in the mechanisms underlying antidepressant efficacy, circulating BDNF cannot be considered a direct surrogate of central nervous system neuroplasticity. Peripheral concentrations may reflect contributions from multiple tissues and physiological processes, thereby limiting their specificity as indicators of neuronal adaptation following brain stimulation. Furthermore, clinical improvement in the absence of measurable changes in peripheral BDNF challenges the assumption that increased circulating BDNF is a necessary component of antidepressant response. Establishing its clinical relevance therefore requires not only identifying treatment-associated changes in BDNF concentrations but also demonstrating reproducible associations with clinically meaningful outcomes while accounting for potential confounding factors [21].

Taken together, the available evidence supports the biological plausibility of peripheral BDNF as a treatment-related biomarker while underscoring the limitations that currently restrict its clinical application. Although brain stimulation therapies may influence circulating BDNF concentrations, these changes are not consistently associated with improvements in depressive symptoms. Persistent methodological heterogeneity, biological variability, and uncertainty regarding the relationship between peripheral and central neurotrophic processes complicate the interpretation of existing findings [22]. Accordingly, peripheral BDNF should be regarded as a promising but insufficiently validated biomarker rather than an established indicator of antidepressant efficacy. Future investigations should prioritize standardized BDNF assessment, longitudinal biological sampling, clearly defined clinical response criteria, and rigorous analytical approaches to determine whether changes in peripheral BDNF provide clinically meaningful information beyond conventional measures of antidepressant response.

This systematic review aims to synthesize the available evidence on changes in peripheral BDNF concentrations following ECT and rTMS in adults with MDD and to evaluate their association with antidepressant outcomes. Specifically, the review examines serum and plasma BDNF dynamics in relation to depressive symptom severity, clinical response, and remission, while identifying methodological factors that may contribute to heterogeneity and influence the interpretation of peripheral BDNF as a potential biomarker of treatment response.

## Review

Methods

This systematic review was conducted in accordance with the Preferred Reporting Items for Systematic Reviews and Meta-Analyses (PRISMA) 2020 guidelines to promote methodological rigor, transparency, and reproducibility throughout the review process. The review protocol was prospectively registered in the International Prospective Register of Systematic Reviews (PROSPERO) (registration number: CRD420261287863).

Eligibility Criteria

The eligibility criteria were developed using the Population, Intervention, Comparator, and Outcome (PICO) framework.

Inclusion criteria: Studies were eligible for inclusion if they met the following predefined criteria: (i) included adults aged 18 years or older with a primary diagnosis of MDD, established according to the Diagnostic and Statistical Manual of Mental Disorders, Fifth Edition, Text Revision (DSM-5-TR) [23] or the International Classification of Diseases, Eleventh Revision (ICD-11) [24]; (ii) investigated the effects of ECT or rTMS; (iii) measured peripheral BDNF concentrations in serum or plasma both at baseline and after treatment; (iv) assessed depressive symptom severity or clinical response using validated instruments, including the Hamilton Depression Rating Scale (HDRS), Montgomery-Åsberg Depression Rating Scale (MADRS), or Beck Depression Inventory (BDI); and (v) employed a randomized controlled trial, non-randomized controlled study, or longitudinal pre-post study design. Eligible comparisons included sham stimulation, usual care, active treatments, no-treatment controls, and within-group assessments conducted before and after treatment. The primary outcome was the change in peripheral BDNF concentrations following brain stimulation, while secondary outcomes comprised changes in depressive symptom severity and the relationship between peripheral BDNF changes and clinical response.

Exclusion criteria: Studies were excluded if they involved children or adolescents, animal or in vitro models, or participants with a primary psychiatric diagnosis other than MDD. Narrative reviews, systematic reviews, meta-analyses, editorials, letters to the editor, case reports, case series involving fewer than five participants, and conference abstracts without sufficient data for extraction were also excluded. Additionally, studies investigating exclusively pharmacological or psychotherapeutic interventions without a brain stimulation component were considered ineligible. Studies that did not report peripheral brain-derived neurotrophic factor (BDNF) concentrations in serum or plasma at both baseline and following treatment were likewise excluded.

Study Selection

A comprehensive literature search was conducted in PubMed/MEDLINE (Medical Literature Analysis and Retrieval System Online), PsycINFO, and LILACS (Latin America and the Caribbean Literature on Health Sciences), supplemented by backward and forward citation tracking and searches of clinical trial registries to identify additional potentially eligible studies. The search was restricted to publications in English and Spanish published between January 2016 and July 2026 to maximize the identification of relevant evidence within the predefined timeframe. Controlled vocabulary terms, including Medical Subject Headings (MeSH) where applicable, and free-text keywords related to MDD, BDNF, ECT, transcranial magnetic stimulation, brain stimulation, neuroplasticity, and clinical response were combined using the Boolean operators “AND” and “OR.” Search terms and their combinations were adapted to the indexing systems and search functionalities of each database to optimize the retrieval of relevant studies. The complete search strategy is presented in Table 1. 

**Table 1:** Literature search strategy MEDLINE: Medical Literature Analysis and Retrieval System Online; LILACS: Latin America and the Caribbean Literature on Health Sciences

Category	Details
Databases searched	PubMed, MEDLINE, PsycINFO, LILACS
Time frame	January 2010 – July 2026
Language	English and Spanish
Search strategy	#1 AND #2 AND #3
#1 (Population)	"Major depressive disorder" OR "Depression" OR "Depressive disorder" OR "Treatment-resistant depression"
#2 (Intervention)	"Electroconvulsive therapy" OR "ECT" OR "Transcranial magnetic stimulation" OR "TMS" OR "Repetitive transcranial magnetic stimulation" OR "rTMS" OR "Transcranial direct current stimulation" OR "tDCS" OR "Non-invasive brain stimulation"
#3 (Outcome)	"Brain-derived neurotrophic factor" OR "BDNF"

All records identified through the electronic database searches and supplementary sources were imported into reference management software, where duplicate records were identified and removed before screening. Two reviewers (ICO and AL) independently screened titles and abstracts against the predefined eligibility criteria. Records considered potentially relevant were subsequently retrieved for full-text assessment, and their eligibility was independently evaluated by both reviewers. Disagreements arising at any stage of the selection process were resolved through discussion and consensus; when consensus could not be achieved, a third reviewer (CDG) was consulted.

Data Extraction

Data extraction was independently conducted by two reviewers (ICO and AL) using a standardized extraction form. Extracted information included study characteristics (first author, publication year, country, and study design); participant characteristics (sample size, age, sex, diagnostic criteria, baseline depression severity, and treatment resistance); and intervention characteristics (brain stimulation modality, stimulation parameters, treatment duration, and number of sessions). Biological variables comprised peripheral BDNF concentrations measured in serum or plasma before and after treatment, laboratory quantification methods, and timing of blood sampling. Clinical outcomes included changes in depressive symptom severity assessed using validated rating scales, as well as reported treatment response or remission. Particular attention was given to the direction and magnitude of peripheral BDNF changes and their association with antidepressant outcomes. When available, reported correlations and other measures of association between BDNF dynamics and clinical response were systematically recorded. Discrepancies between reviewers were resolved through discussion and consensus, with consultation of a third reviewer (CDG) when necessary.

Risk of Bias and Quality Assessment

The methodological quality and risk of bias of the included studies were evaluated according to study design. Randomized controlled trials were assessed using the Cochrane Risk of Bias Tool, version 2 (RoB 2), which examines potential bias arising from the randomization process, deviations from intended interventions, missing outcome data, outcome measurement, and selection of reported results [25]. Non-randomized and observational studies were assessed using the Newcastle-Ottawa Scale (NOS), where applicable, considering participant selection, comparability, and outcome or exposure assessment [26]. The certainty of the body of evidence for each relevant outcome was evaluated using the Grading of Recommendations Assessment, Development and Evaluation (GRADE) framework [27]. All assessments were independently performed by two reviewers (ICO and AL), and disagreements were resolved through discussion and consensus.

Data Synthesis

Quantitative synthesis was initially considered for studies demonstrating sufficient clinical and methodological comparability. The proposed analytical approach included random-effects meta-analysis models, with effect estimates expressed as standardized mean differences (SMDs) and corresponding 95% confidence intervals (CIs). Statistical heterogeneity would have been assessed using Cochran's Q test and the I² statistic, with subgroup analyses considered according to brain stimulation modality (ECT versus rTMS) and biological specimen type (serum versus plasma). However, an assessment of the included studies revealed substantial clinical and methodological heterogeneity that precluded meaningful quantitative pooling. Specifically, differences in patient populations, baseline depression severity, treatment resistance, stimulation protocols, concomitant medications, biological matrices used for peripheral BDNF measurement, sampling intervals, and clinical outcome measures limited the comparability of reported findings. Furthermore, variations in the reporting of baseline and post-treatment BDNF concentrations, effect estimates, and definitions of clinical response prevented the identification of sufficiently comparable data for a reliable pooled analysis. Consequently, no meta-analysis was performed, and statistical heterogeneity measures and subgroup analyses were not calculated.

Instead, a structured narrative synthesis was conducted. Changes in peripheral BDNF concentrations were examined separately from their associations with antidepressant response, considering the direction and consistency of findings, differences in study methodologies, and clinical outcomes across ECT and rTMS studies. This approach allowed the available evidence to be summarized without generating potentially misleading pooled estimates.

Results

Study Selection

The literature search identified 92 records across PubMed (n = 43), PsycINFO (n = 26), LILACS (n = 14), and MEDLINE (n = 9). Before screening, nine duplicate records and eight records excluded for other reasons were removed, leaving 75 records for title and abstract screening. Of these, 18 were excluded, and 57 reports were sought for retrieval. Twenty-three reports could not be retrieved, leaving 34 full-text articles for eligibility assessment. Following full-text evaluation, 23 reports were excluded due to ineligible outcomes (n = 7), interventions that did not meet the predefined inclusion criteria (n = 8), or language restrictions (n = 8). Ultimately, 11 studies met the eligibility criteria and were included in the systematic review. The study selection process is illustrated in the PRISMA 2020 flow diagram (Figure 1).

**Figure 1:**
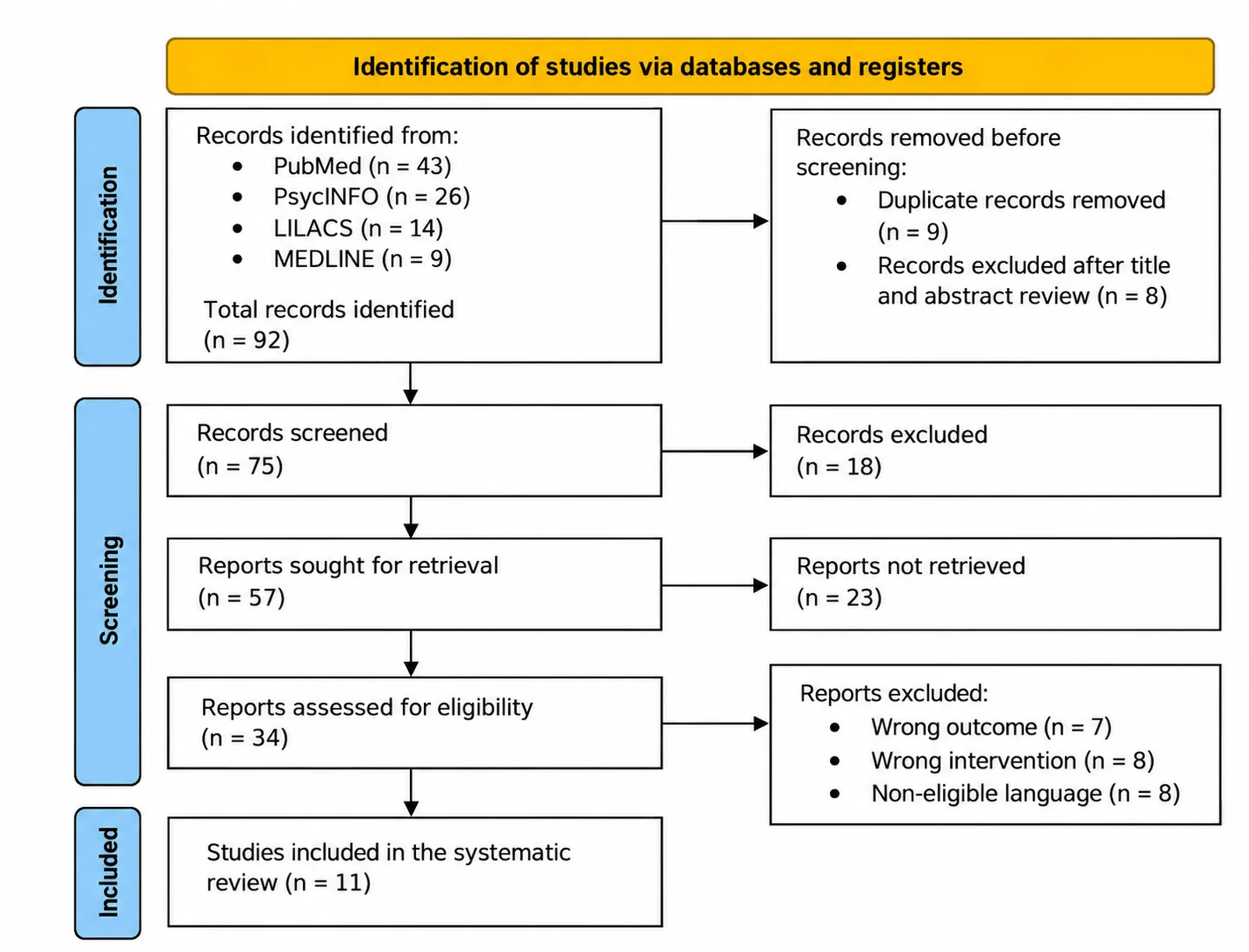
PRISMA flow diagram indicating the study selection and inclusion process PRISMA: Preferred Reporting Items for Systematic Reviews and Meta-Analyses

Characteristics of Included Studies

Table 2 summarizes the main characteristics of the 11 studies [28-38] included in this systematic review, including study design, participant demographics, brain stimulation modalities, and biological and clinical outcomes. The studies investigated changes in peripheral BDNF concentrations following brain stimulation therapies in adults with MDD. Particular attention was given to differences in stimulation protocols, serum or plasma BDNF measurements, depressive symptom severity, and the reported associations between peripheral BDNF dynamics and antidepressant response.

**Table 2:** Summary of included studies on peripheral BDNF changes following brain stimulation in MDD BDNF: brain-derived neurotrophic factor; ECT: electroconvulsive therapy; MDD: major depressive disorder; rTMS: repetitive transcranial magnetic stimulation; TNF-α: tumor necrosis factor-alpha

Study (Author, Year)	Study design	Population	Outcomes relevant to the systematic review
Vanicek et al. (2019) [28]	Prospective longitudinal study	24 adults with MDD undergoing acute ECT; five received continuation ECT	Significant increases in serum BDNF following ECT; no significant changes in plasma BDNF or association with antidepressant response.
Xie et al. (2023) [29]	Longitudinal observational study with healthy controls	40 patients with treatment-resistant MDD and 35 healthy controls; 34 completed post-ECT assessment	Increased plasma BDNF following ECT; lower baseline BDNF associated with greater depressive symptom severity.
Valiuliene et al. (2021) [30]	Prospective interventional study with healthy controls	25 adults with treatment-resistant unipolar depression receiving rTMS	Increased serum BDNF following rTMS, accompanied by reductions in depressive symptoms; baseline TNF-α identified as a potential predictor of response.
Mindt et al. (2020) [31]	Longitudinal pre–post study	9 adults with severe treatment-resistant MDD undergoing ECT	No significant changes in serum BDNF or association with depressive symptom improvement; cerebrospinal fluid BDNF increased significantly.
Permoda-Pachuta et al. (2023) [32]	Longitudinal observational study	29 patients with treatment-resistant unipolar depression receiving ECT, extracted from a mixed diagnostic cohort	No significant changes in serum BDNF in the unipolar subgroup; the association with clinical improvement remained inconclusive.
Sorri et al. (2018) [33]	Prospective longitudinal study	30 adults with MDD receiving bilateral ECT	No significant overall changes in serum BDNF; transient decreases in plasma BDNF; no consistent association with remission.
Salehi et al. (2016) [34]	Three-arm randomized interventional study	60 adults with treatment-resistant MDD assigned to ECT, ECT plus aerobic exercise, or aerobic exercise alone	Increased plasma BDNF and reduced depressive symptoms across groups; greatest improvements with combined treatment; no significant BDNF–symptom association.
Tong et al. (2021) [35]	Randomized, double-blind, sham-controlled trial	120 adults with MDD randomized to active or sham rTMS	Improved depressive symptoms and cognitive functioning following active rTMS; no significant between-group differences in serum BDNF.
Bouckaert et al. (2016) [36]	Prospective longitudinal study	88 older adults with severe unipolar MDD receiving ECT	Serum BDNF remained unchanged despite improvements in depressive symptoms and hippocampal volume; no association with antidepressant response.
Bilgen et al. (2014) [37]	Prospective longitudinal study with healthy controls	30 adults with treatment-resistant MDD receiving ECT and 30 healthy controls	Significant increases in serum BDNF following ECT, measured at baseline, clinical response, and treatment completion.
Gedge et al. (2012) [38]	Prospective, single-blind comparative study	29 adults with treatment-resistant MDD receiving ECT (n = 11) or rTMS (n = 18)	No significant changes in serum BDNF following ECT or rTMS despite clinical improvement; limited support for BDNF as a response biomarker.

Table 3 summarizes the principal findings and conclusions of the 11 studies selected for this systematic review, focusing on changes in peripheral BDNF concentrations following brain stimulation therapies and their relationship with antidepressant response in adults with MDD. Overall, the findings demonstrate substantial variability in serum and plasma BDNF responses to ECT and rTMS. Although several studies reported increased peripheral BDNF concentrations following treatment [28-30,34,36], others observed no significant changes despite improvements in depressive symptoms [31,33,35,36,38]. Furthermore, associations between BDNF dynamics and clinical outcomes were inconsistent, suggesting that peripheral BDNF may reflect certain treatment-related biological processes without reliably predicting antidepressant response. These findings highlight the need for standardized sampling procedures, consistent stimulation protocols, and adequately powered longitudinal investigations to clarify the clinical utility of peripheral BDNF as a biomarker of treatment response. 

**Table 3:** Summary of results and conclusions of included studies on peripheral BDNF changes following brain stimulation in MDD BDNF: brain-derived neurotrophic factor; ECT: electroconvulsive therapy; MDD: major depressive disorder; rTMS: repetitive transcranial magnetic stimulation; TNF-α: tumor necrosis factor-alpha

Study (Author, Year)	Results	Conclusions
Vanicek et al. [28]	Serum BDNF concentrations increased following acute ECT, whereas plasma BDNF remained unchanged. No statistically significant association was identified between serum BDNF concentrations and depressive symptom severity or treatment response.	ECT may induce changes in serum BDNF without corresponding alterations in plasma concentrations. These findings do not support peripheral BDNF as a reliable indicator of antidepressant response.
Xie et al. [29]	Plasma BDNF concentrations were lower in patients with treatment-resistant depression than in healthy controls at baseline and increased following ECT. Lower baseline BDNF was associated with greater depressive symptom severity.	ECT may modify peripheral BDNF concentrations in treatment-resistant depression. However, the relationship between treatment-induced BDNF changes and clinical response requires further investigation.
Valiuliene et al. [30]	Serum BDNF concentrations increased following rTMS, accompanied by improvements in depressive symptoms. Baseline TNF-α, rather than BDNF, was identified as a potential predictor of treatment success.	rTMS may influence peripheral neurotrophic activity in patients with treatment-resistant unipolar depression. Nevertheless, the predictive value of serum BDNF for antidepressant response remains uncertain.
Mindt et al. [31]	Serum BDNF concentrations did not change significantly following ECT, despite clinical improvement. In contrast, cerebrospinal fluid BDNF increased significantly, without a corresponding association between serum BDNF changes and depressive symptom reduction.	Peripheral and central BDNF responses to ECT may differ, suggesting that serum BDNF does not necessarily reflect central neuroplastic changes or antidepressant response.
Permoda-Pachuta et al. [32]	Serum BDNF concentrations showed no statistically significant changes following ECT in the unipolar depression subgroup. The reported relationship between BDNF concentrations and clinical improvement was not established specifically for this subgroup.	Serum BDNF changes following ECT appear inconsistent in treatment-resistant unipolar depression. The available subgroup findings do not establish BDNF as a reliable biomarker of clinical response.
Sorri et al. [33]	Serum BDNF concentrations remained largely unchanged during ECT, while plasma BDNF showed a transient decrease during treatment. Changes in peripheral BDNF were not consistently associated with clinical remission.	Peripheral BDNF concentrations may fluctuate during ECT without reflecting sustained antidepressant improvement, limiting their utility as standalone biomarkers of remission.
Salehi et al. [34]	Plasma BDNF concentrations increased following ECT, aerobic exercise, and combined treatment, alongside reductions in depressive symptom severity. The combined intervention produced greater improvements, although BDNF changes were not significantly associated with depressive symptoms.	ECT and aerobic exercise may influence peripheral BDNF concentrations through treatment-related neurobiological mechanisms. However, increased BDNF does not necessarily correspond to greater antidepressant response.
Tong et al. [35]	Active rTMS improved depressive symptoms and selected cognitive outcomes compared with sham stimulation. However, no statistically significant between-group differences were observed in serum BDNF concentrations.	The antidepressant and cognitive benefits of rTMS may occur independently of measurable changes in peripheral BDNF, limiting its value as a treatment-response biomarker.
Bouckaert et al. [36]	Serum BDNF concentrations remained unchanged following ECT despite significant improvements in depressive symptoms and increases in hippocampal volume. No significant association was identified between BDNF changes, hippocampal volume, and clinical improvement.	ECT-related clinical and structural brain changes may occur without corresponding alterations in serum BDNF, suggesting that peripheral measurements do not reliably capture central neuroplasticity or antidepressant response.
Bilgen et al. [37]	Serum BDNF concentrations increased significantly during ECT, with higher levels observed at clinical response and treatment completion compared with baseline.	ECT may enhance peripheral BDNF concentrations in treatment-resistant MDD, supporting a potential relationship with treatment-related neurotrophic processes. However, the clinical predictive value of these changes remains uncertain.
Gedge et al. [38]	No statistically significant changes in serum BDNF concentrations were observed following either ECT or rTMS, despite improvements in depressive symptoms.	Antidepressant improvement following ECT or rTMS may occur independently of changes in circulating BDNF, providing limited support for serum BDNF as a reliable biomarker of treatment response.

Table 4 presents the risk of bias assessment of the 11 studies included in this systematic review, conducted using design-specific methodological appraisal tools. Randomized controlled trials were evaluated using the RoB 2, whereas observational and non-randomized studies were assessed using the NOS. The methodological appraisal considered participant selection, comparability, potential confounding, outcome measurement, and completeness of follow-up. Risk of bias was provisionally classified as low, moderate, or moderate-to-high according to the methodological limitations identified in each study. The certainty of evidence for each outcome was assessed using the GRADE framework, with the findings and proposed certainty ratings summarized in Table 5.

**Table 4:** Risk of bias assessment of included studies The Cochrane Risk of Bias 2 (RoB 2) tool [25] was used for randomized controlled trials, whereas a modified version of the Newcastle–Ottawa Scale (NOS) [26] was applied to observational studies, including uncontrolled longitudinal pre–post studies. All risk-of-bias assessments were completed independently by two reviewers, and the classifications presented represent the final methodological evaluations.

Study (Author, Year)	Study design	Risk of bias	Tool used	Justification
Vanicek et al. (2019) [28]	Prospective longitudinal study	Moderate	NOS	Uncontrolled pre–post design, small sample size, and potential confounding by concomitant treatments.
Xie et al. (2023) [29]	Longitudinal observational study with healthy controls	Moderate	NOS	Non-randomized participant selection, potential baseline differences, and incomplete post-treatment follow-up.
Valiuliene et al. (2021) [30]	Prospective interventional study with healthy controls	Moderate	NOS	Non-randomized treatment allocation, limited sample size, and potential confounding by concomitant therapies.
Mindt et al. (2020) [31]	Longitudinal pre–post study	Moderate–High	NOS	Very small sample size, absence of a comparison group, and limited ability to attribute BDNF changes specifically to ECT.
Permoda-Pachuta et al. (2023) [32]	Longitudinal observational study	Moderate–High	NOS	Diagnostic heterogeneity, subgroup-specific analyses, and potential confounding affecting interpretation of unipolar depression outcomes.
Sorri et al. (2018) [33]	Prospective longitudinal study	Moderate	NOS	Standardized biomarker measurements, but limited sample size, absence of a comparator group, and potential residual confounding.
Salehi et al. (2016) [34]	Three-arm randomized controlled trial	Low	RoB 2 (Cochrane)	Randomized allocation reported; allocation concealment, blinding, deviations from interventions, and selective reporting require verification.
Tong et al. (2021) [35]	Randomized double-blind sham-controlled trial	Low	RoB 2 (Cochrane)	Randomized sham-controlled design and blinding; allocation procedures, missing data, and selective outcome reporting require verification.
Bouckaert et al. (2016) [36]	Prospective longitudinal study	Moderate	NOS	Repeated outcome measurements, but absence of an untreated comparator and potential confounding in associations between BDNF and clinical response.
Bilgen et al. (2014) [37]	Prospective longitudinal study with healthy controls	Moderate	NOS	Healthy control group and repeated BDNF measurements, but non-randomized design and potential residual confounding.
Gedge et al. (2012) [38]	Prospective single-blind comparative study	Moderate–High	NOS	Small intervention groups, non-randomized treatment allocation, potential baseline differences, and limited statistical precision.

**Table 5:** Summary of findings and proposed GRADE certainty of evidence for BDNF changes following ECT and rTMS in MDD GRADE certainty levels: High (⊕⊕⊕⊕); Moderate (⊕⊕⊕◯); Low (⊕⊕◯◯); Very low (⊕◯◯◯) BDNF, brain-derived neurotrophic factor; CSF, cerebrospinal fluid; ECT, electroconvulsive therapy; GRADE, Grading of Recommendations Assessment, Development and Evaluation; MDD, major depressive disorder; rTMS, repetitive transcranial magnetic stimulation

Outcome	Studies (authors)	Summary of findings	Consistency of findings	Proposed GRADE certainty
Serum BDNF following ECT	Vanicek et al. [28]; Mindt et al. [31]; Permoda-Pachuta et al. [30]; Sorri et al.. [33]; Bouckaert et al. [36]; Bilgen et al. [37]	Several prospective studies reported statistically significant increases in serum BDNF following ECT, supporting a treatment-associated neurotrophic response. Other studies reported no significant changes.	Concordant significant increases in several cohorts, with incomplete replication across all studies.	⊕⊕⊕◯ Moderate
Plasma BDNF following ECT	Vanicek et al. [28]; Xie et al. [29]; Sorri et al. [33]; Salehi et al. [34]	Several studies reported significant increases in plasma BDNF after ECT, sometimes accompanied by reductions in depressive symptoms. Other studies reported unchanged concentrations or transient reductions.	Concordant increases in studies reporting positive effects, with some variation in direction and timing.	⊕⊕⊕◯ Moderate
Serum BDNF following rTMS	Valiuliene et al. [30]; Tong et al. [35]; Gedge et al. [38]	One study reported significantly increased serum BDNF, while others found clinical improvement without significant biomarker changes.	Variable findings; BDNF increases were not consistently reproduced.	⊕⊕⊕◯ Moderate
Active versus sham rTMS	Tong et al. [35]	Active rTMS improved depressive symptoms and selected cognitive outcomes without significant between-group differences in serum BDNF.	Between-study consistency not assessable; one randomized trial.	⊕⊕⊕◯ Moderate
Randomized intervention comparisons	Salehi et al. [34]	ECT and aerobic exercise were associated with improvements in depressive symptoms and plasma BDNF; the combined intervention showed the greatest reported improvements.	Between-study consistency not assessable; one randomized trial.	⊕⊕⊕◯ Moderate
Baseline BDNF and depressive severity	Xie et al. [29]	Lower baseline plasma BDNF was associated with greater depressive symptom severity.	Between-study consistency not assessable; one observational cohort.	⊕⊕◯◯ Low
BDNF changes and antidepressant response	Vanicek [28]; Valiuliene [30]; Mindt [31]; Permoda-Pachuta [32]; Sorri [33]; Salehi [34]; Bouckaert [36]; Gedge [38]	Although BDNF increases accompanied clinical improvement in some studies, biomarker changes were not consistently associated with antidepressant response.	Associations varied across studies.	⊕⊕◯◯ Low
CSF BDNF following ECT	Mindt [31]	CSF BDNF increased significantly following ECT despite unchanged serum concentrations.	Between-study consistency not assessable; one small longitudinal cohort.	⊕⊕◯◯ Low

Study Findings

This systematic review synthesized evidence from 11 studies examining changes in peripheral BDNF concentrations following ECT and rTMS in adults with MDD. The evidence comprised two randomized controlled trials [34,35] and nine prospective or non-randomized investigations [28-33,36-38], with substantial variation in study design, sample size, stimulation protocols, biological sampling procedures, and clinical outcomes. Across studies, treatment-associated changes in peripheral BDNF were inconsistent, ranging from significant increases in serum or plasma concentrations to unchanged or transiently reduced levels. Furthermore, alterations in circulating BDNF were not consistently associated with reductions in depressive symptom severity, treatment response, or remission.

In a prospective longitudinal study involving 24 patients with MDD undergoing ECT, mean serum BDNF concentrations increased from 24.68 ± 14.40 ng/mL at baseline to 33.04 ± 14.11 ng/mL one day after the final treatment session (adjusted p = 0.013), 37.03 ± 10.29 ng/mL after one week (adjusted p < 0.001), and 41.05 ± 10.67 ng/mL after one month (adjusted p = 0.008) [28]. In contrast, plasma BDNF concentrations did not change significantly (p > 0.1), and serum BDNF changes were not significantly associated with clinical response. A separate longitudinal investigation involving 40 patients with treatment-resistant depression and 35 healthy controls identified lower baseline plasma BDNF concentrations in the patient group and increased concentrations following ECT [29]. Post-treatment biomarker measurements were available for 34 patients; however, the reported findings did not establish the predictive accuracy of plasma BDNF changes for antidepressant response. Evidence from rTMS was also suggestive of treatment-related neurotrophic alterations. In a prospective investigation involving 25 patients with treatment-resistant depression, serum BDNF concentrations increased following stimulation, while lower pretreatment TNF-α concentrations were associated with more favorable clinical outcomes [30]. These findings distinguished treatment-related BDNF changes from the potential prognostic contribution of baseline inflammatory markers.

In contrast, several investigations reported no significant peripheral BDNF increases following ECT. A preliminary longitudinal study involving nine patients found increased cerebrospinal fluid BDNF concentrations without corresponding significant changes in serum concentrations, demonstrating discordant findings across biological compartments [31]. Another observational investigation examining ECT in treatment-resistant mood disorders reported no significant pre-to-post-treatment change in serum BDNF within the unipolar depression subgroup (p = 0.974) [32]. An exploratory analysis also identified a weak negative correlation between baseline BDNF concentrations and the percentage change in HDRS scores (Spearman's ρ = −0.286, p = 0.036); however, because the broader study population included different mood disorder diagnoses, this association cannot be attributed specifically to unipolar depression without confirmation of the subgroup analysis. In a prospective study of 30 patients with MDD, mean MADRS scores decreased from 31.6 ± 7.2 at baseline to 11.3 ± 7.5 following ECT, with 20 participants (66.7%) achieving remission [33]. Despite substantial symptomatic improvement, serum BDNF concentrations remained statistically unchanged. Plasma BDNF exhibited a transient reduction two hours after the fifth ECT session (p = 0.038; Cohen's d = 0.45), but neither baseline concentrations nor longitudinal biomarker changes were significantly associated with remission.

Randomized controlled evidence further demonstrated that peripheral neurotrophic changes and clinical outcomes did not consistently follow parallel trajectories. In a three-arm randomized trial involving 60 patients with MDD, ECT, aerobic exercise, and their combination were evaluated over four weeks alongside standard antidepressant pharmacotherapy [34]. Plasma BDNF concentrations increased across the three treatment groups, accompanied by reductions in depressive symptom severity. The combined intervention was associated with greater symptomatic improvement and higher remission rates than either intervention alone, with a significant relationship between treatment allocation and remission (χ² = 20.03, p < 0.001). However, inconsistencies in the original trial's reporting prevented a definitive determination of which intervention group exhibited the greatest increase in plasma BDNF concentrations. Consequently, the comparative biomarker findings were classified as uncertain in the narrative synthesis, and no definitive conclusions were drawn regarding the relative effects of ECT, aerobic exercise, or combined treatment on plasma BDNF levels. Nevertheless, plasma BDNF concentrations were not significantly associated with depressive symptom severity, and the observed increases did not establish a biomarker-specific explanation for the differences in clinical outcomes. In a randomized, double-blind, sham-controlled trial involving 120 patients with MDD, active and sham rTMS were compared over 20 treatment sessions administered during four weeks [35]. Active stimulation, delivered at 10 Hz over the left dorsolateral prefrontal cortex, was associated with greater improvements in facial emotion identification (F = 18.09, p < 0.001) and Hinting Task performance (F = 5.02, p = 0.026). However, these cognitive outcomes were distinct from the principal biomarker question, and the available findings did not demonstrate a consistent between-group difference in serum BDNF concentrations or establish its relationship with antidepressant response.

Further longitudinal evidence supported the absence of a reproducible correspondence between peripheral BDNF changes and clinical improvement. In a prospective investigation involving 88 older adults with severe unipolar depression receiving ECT, depressive symptom severity decreased significantly, accompanied by increases in hippocampal volume [36]. Neuroimaging data were available for 66 participants shortly after treatment and for 23 at longer-term follow-up. Despite these clinical and structural changes, serum BDNF concentrations remained unchanged at the principal post-treatment assessments, and no significant parallel relationship was identified among peripheral BDNF trajectories, depressive symptom improvement, and hippocampal volume changes. Conversely, a prospective study involving 30 patients with treatment-resistant MDD and 30 healthy controls reported significantly increased serum BDNF concentrations following ECT (p < 0.05), together with reductions in HDRS scores [37]. However, serum BDNF concentrations were not significantly correlated with depressive symptom severity (p > 0.05), limiting the evidence for a direct correspondence between circulating BDNF and clinical improvement. Similarly, a comparative investigation involving 29 patients with treatment-resistant MDD, including 11 receiving ECT and 18 receiving rTMS, documented improvements in depressive symptoms without significant increases in serum BDNF concentrations measured before and after treatment [38].

Across the included studies, the interpretation of peripheral BDNF changes was complicated by differences in biological matrices and measurement intervals. Discordant serum and plasma findings were observed within individual ECT investigations [28,33], while differences between cerebrospinal fluid and serum measurements were also documented [29]. In addition, statistically significant increases in circulating BDNF did not consistently correspond to improvements in depressive symptom severity or remission [28,34,37]. Conversely, clinical improvement was reported in several investigations without significant peripheral BDNF increases [33,35,37,38]. These findings were derived from studies with heterogeneous treatment protocols, sample sizes, concomitant therapies, and follow-up schedules, limiting direct comparisons across interventions and biological outcomes.

Overall, the included evidence demonstrated heterogeneous peripheral BDNF responses following ECT and rTMS in adults with MDD. Although increases in circulating BDNF were documented in several investigations, the direction and magnitude of these changes were not consistently associated with antidepressant response or remission. Accordingly, the available findings do not establish peripheral BDNF as a validated standalone biomarker for predicting or monitoring clinical outcomes following brain stimulation.

Due to substantial clinical and methodological heterogeneity across the included studies, particularly regarding stimulation protocols, biological matrices, BDNF measurement timing, and clinical outcomes, a meta-analysis was not performed. Instead, the findings were integrated through a structured narrative synthesis to avoid potentially misleading pooled estimates.

Discussion

The present systematic review synthesizes evidence from 11 studies investigating changes in peripheral BDNF concentrations following ECT and rTMS in adults with MDD. The findings reveal substantial variability in peripheral neurotrophic responses despite clinically meaningful improvements in depressive symptom severity, particularly following ECT. Although several investigations reported increased serum or plasma BDNF concentrations after treatment, others demonstrated considerable symptomatic improvement without corresponding changes in circulating BDNF [28-39]. This discrepancy highlights an important distinction between the clinical effectiveness of brain stimulation and the validity of peripheral BDNF as a biomarker of antidepressant response. Furthermore, the heterogeneous findings suggest that circulating BDNF concentrations may reflect treatment-associated biological processes without consistently capturing the magnitude or trajectory of clinical improvement.

The clinical relevance of this dissociation is particularly evident in a prospective investigation involving 30 patients with MDD undergoing ECT [33]. Mean MADRS scores decreased from 31.6 ± 7.2 at baseline to 11.3 ± 7.5 following treatment, corresponding to an absolute reduction of 20.3 points and an approximately 64% reduction in mean depressive symptom severity. Furthermore, 20 participants (66.7%) achieved remission. Despite these substantial clinical improvements, serum BDNF concentrations remained statistically unchanged, while plasma BDNF exhibited a transient reduction during treatment without a consistent association with remission. Similarly, a longitudinal investigation involving older adults with severe unipolar depression demonstrated improvements in depressive symptom severity and increases in hippocampal volume without corresponding significant changes in serum BDNF [36]. These findings are clinically important because they demonstrate that marked symptomatic improvement may occur in the absence of measurable increases in peripheral BDNF. However, the absence of a significant peripheral biomarker response does not exclude the involvement of central BDNF-dependent neuroplastic mechanisms in the therapeutic effects of ECT.

Previous quantitative syntheses provide an important context for interpreting these findings. An updated systematic review and meta-analysis involving 28 studies and 778 participants identified a modest increase in peripheral BDNF following ECT (Hedges' g = 0.28; 95% CI, 0.10-0.46), accompanied by substantial between-study heterogeneity (I² = 67.64%) [35]. In contrast, the pooled reduction in depressive symptom severity was considerably larger (Hedges' g = −3.72; 95% CI, −4.23 to −3.21). Although these effect sizes represent different outcomes and cannot be directly compared to establish mechanistic independence, they illustrate that the magnitude of symptomatic improvement is not necessarily mirrored by changes in circulating BDNF [39]. A separate meta-analysis restricted to patients with MDD similarly reported increased BDNF concentrations following ECT (SMD = 0.56; 95% CI, 0.17-0.96), although substantial heterogeneity was observed (I² = 73%) [40]. These findings suggest that ECT-associated changes in peripheral BDNF may be detectable at the group level, but their relationship with individual clinical outcomes remains insufficiently established.

More recent evidence further emphasizes the importance of biological sampling and methodological variability. A 2026 meta-analysis examining ECT-associated BDNF changes in MDD reported increased concentrations in both serum and plasma, while identifying substantial heterogeneity among serum studies (I² = 86%) and no detected statistical heterogeneity among plasma studies (I² = 0%) [41]. Anesthetic type and blood-sampling time were also identified as potential moderators of neurotrophic variability. These observations provide a relevant context for the discordant serum and plasma responses documented within individual investigations included in the present review [28,33]. Nevertheless, the absence of detected heterogeneity in a relatively small subgroup of plasma studies does not establish that plasma measurements are intrinsically more accurate or clinically informative. Furthermore, study-level moderator associations should not be interpreted as evidence of causal relationships between anesthetic selection, sampling procedures, and individual neurotrophic responses.

A particularly relevant finding concerns the comparative effects of ECT, aerobic exercise, and their combination. In a three-arm randomized trial involving 60 patients with MDD, ECT, aerobic exercise, and combined treatment were associated with reductions in depressive symptom severity and increases in plasma BDNF over four weeks [34]. Notably, the combined intervention was associated with greater symptomatic improvement and higher remission rates than either intervention alone. However, plasma BDNF concentrations were not significantly associated with depressive symptom severity. This finding is particularly informative because it distinguishes the clinical benefits of treatment from the biological interpretation of peripheral neurotrophic changes. Although both clinical and biomarker outcomes improved, the available analyses did not establish that changes in BDNF explained the antidepressant effects of either intervention. Moreover, the publication contains inconsistent descriptions of the treatment group exhibiting the greatest increase in plasma BDNF, limiting definitive interpretation of the relative biomarker effects without clarification of the original data.

The interpretation of this trial also requires methodological caution. Its modest sample size, short follow-up, and concomitant selective serotonin reuptake inhibitor treatment limit the attribution of neurotrophic changes exclusively to ECT or exercise [34]. Furthermore, the simultaneous occurrence of increased BDNF concentrations and reduced depressive symptoms does not establish neurotrophic mediation. Demonstrating such mediation would require temporally ordered measurements and formal analyses evaluating whether changes in BDNF account for a meaningful proportion of the treatment effect. Consequently, the findings support further investigation of combined ECT and aerobic exercise as a potentially beneficial therapeutic strategy, but do not establish that peripheral BDNF mediates the additional clinical improvement. This distinction is particularly important because the antidepressant effects of exercise may involve several biological and psychosocial pathways beyond neurotrophic signaling.

The broader literature on physical exercise provides additional context. A systematic review and network meta-analysis including 218 randomized controlled trials and 14,170 participants identified reductions in depressive symptoms across several exercise modalities [42]. Compared with active controls, walking or jogging demonstrated an estimated effect of Hedges' g = −0.62 (95% credible interval, −0.80 to −0.45), while mixed aerobic exercise demonstrated an effect of −0.43 (95% credible interval, −0.61 to −0.24). These findings support the antidepressant effects of structured physical activity, although methodological limitations reduced confidence in several estimates. Importantly, evidence of exercise-related clinical improvement should not be interpreted as direct evidence that circulating BDNF mediates these benefits. Similarly, a systematic review incorporating 36 randomized controlled trials and 2,515 participants reported that different exercise modalities may increase peripheral BDNF concentrations in patients with depression [43]. Nevertheless, exercise also induces cardiovascular, metabolic, inflammatory, and platelet-related physiological responses that may contribute to circulating BDNF independently of central antidepressant mechanisms.

The combined ECT-exercise intervention therefore raises an important mechanistic question regarding whether these treatments engage partially overlapping neuroplastic pathways or produce distinct biological effects that converge on symptomatic improvement. ECT induces therapeutic seizures and widespread neurophysiological adaptations, whereas aerobic exercise involves systemic physiological responses that may influence both central and peripheral neurotrophic signaling. Although both interventions have been associated with changes in BDNF, their effects are not necessarily equivalent in magnitude, timing, or biological origin. Accordingly, the observed clinical benefit of combined treatment cannot be attributed to additive or synergistic central neuroplasticity without appropriate mechanistic investigations. Future studies examining this therapeutic combination should distinguish peripheral biomarker changes from central neurobiological adaptations and evaluate whether neurotrophic responses provide explanatory information beyond clinical outcomes alone.

The interpretation of circulating BDNF is further complicated by differences between peripheral and central biological compartments. Although BDNF participates in neuronal survival, synaptic plasticity, and activity-dependent neural adaptation, blood concentrations are influenced by peripheral sources and platelet-related processes. Serum measurements are particularly sensitive to platelet release during coagulation, whereas plasma concentrations may be affected by residual platelet contamination and preanalytical procedures. Consequently, serum and plasma BDNF should not be regarded as interchangeable measures of central neurotrophic activity. The observation of increased cerebrospinal fluid BDNF without a corresponding serum increase in one included investigation [31], together with clinical and structural brain changes occurring without significant serum BDNF alterations in another [36], further illustrates the limitations of interpreting circulating BDNF as a direct surrogate of central neuroplasticity.

Evidence from non-invasive brain stimulation supports a similarly cautious interpretation. A systematic review and meta-analysis involving eight datasets and 259 participants found no significant overall increase in peripheral BDNF following rTMS or transcranial direct current stimulation (Hedges' g = 0.03; 95% CI, −0.21 to 0.27) [44]. Although this synthesis included more than one stimulation modality, its findings are consistent with the absence of reproducible peripheral BDNF increases in some rTMS investigations included in the present review [35,38]. Nevertheless, differences in stimulation protocols, treatment duration, baseline depression severity, and biological sampling limit direct comparisons across interventions. The available evidence therefore remains insufficient to determine whether differences in peripheral BDNF responses between ECT and rTMS reflect distinct neurobiological mechanisms or methodological variability.

A broader systematic review and meta-analysis of treatment-resistant depression, involving 36 studies and 1,198 participants, identified a small overall increase in peripheral BDNF following treatment, without a significant association between neurotrophic changes and improvement in depressive symptoms [45]. Because this synthesis included both pharmacological and non-pharmacological interventions, its findings cannot be attributed specifically to ECT or rTMS. Nevertheless, they reinforce the distinction between treatment-associated biological responsiveness and clinically useful predictive validity. An increase in mean biomarker concentration does not establish the ability to distinguish responders from nonresponders, predict remission, or monitor individual treatment trajectories. Establishing such utility would require evidence of discrimination, calibration, reproducibility, and incremental predictive performance beyond established clinical variables.

Limitations

Several limitations should be acknowledged. Most included investigations had relatively small samples, and many employed uncontrolled or non-randomized longitudinal designs, limiting causal attribution of BDNF changes to brain stimulation. Differences in baseline depression severity, treatment resistance, stimulation parameters, concomitant medications, biological sampling intervals, and laboratory procedures restricted comparability across studies. Moreover, serum and plasma BDNF measurements were not consistently standardized, and individual investigations frequently lacked sufficient statistical power to detect modest biomarker-outcome associations. The use of different depression rating scales and definitions of response or remission further complicated the synthesis of clinical findings. Although substantial reductions in MADRS and HDRS scores were documented, not all studies formally evaluated minimal clinically important differences or provided comparable remission criteria. Additionally, the predominance of studies measuring total BDNF rather than distinguishing mature BDNF from its precursor may have obscured biologically relevant differences in neurotrophic signaling. Finally, the present review provides a qualitative synthesis and therefore cannot estimate pooled biomarker-response associations or formally examine sources of statistical heterogeneity. Furthermore, the full texts of 23 potentially relevant reports could not be retrieved because they were hosted on country-specific platforms with restricted access. This limitation may have introduced selection bias and reduced the comprehensiveness of the evidence included in this review.

Future Recommendations

Future research should prioritize adequately powered, methodologically rigorous studies that distinguish treatment-related changes in peripheral BDNF from their potential predictive value for antidepressant response. Particular attention should be given to standardized serum and plasma sampling, repeated biomarker measurements, concomitant pharmacotherapy, and clinically meaningful outcomes, including changes in depression rating scales, treatment response, and remission. Furthermore, the potential complementary effects of ECT and aerobic exercise warrant investigation through controlled trials designed to clarify whether their clinical benefits involve shared or distinct neuroplastic mechanisms. Such approaches may help determine whether peripheral BDNF provides clinically useful information beyond established measures of depressive symptom severity.

## Conclusions

This systematic review indicates that ECT and rTMS are associated with heterogeneous and inconsistent changes in peripheral BDNF concentrations in adults with MDD. Importantly, substantial reductions in depressive symptom severity and clinically meaningful remission rates were observed following ECT, even in the absence of significant changes in circulating BDNF. Although increased peripheral BDNF has been reported following brain stimulation, these changes did not consistently correlate with antidepressant response or remission. Preliminary findings also suggest that combining ECT with aerobic exercise may enhance clinical outcomes, without establishing BDNF as a mediator of these benefits. Collectively, these findings challenge the clinical utility of peripheral BDNF as a standalone biomarker of antidepressant response and highlight the need to distinguish treatment-induced neurotrophic changes from clinically relevant improvements in depression.

## References

[1] Cui L, Li S, Wang S (2024). Major depressive disorder: hypothesis, mechanism, prevention and treatment. Signal Transduct Target Ther.

[2] Greenberg P, Chitnis A, Louie D (2023). The economic burden of adults with major depressive disorder in the United States (2019). Adv Ther.

[3] Trivedi MH, Rush AJ, Wisniewski SR (2006). Evaluation of outcomes with citalopram for depression using measurement-based care in STAR*D: implications for clinical practice. Am J Psychiatry.

[4] Halaris A, Sohl E, Whitham EA (2021). Treatment-resistant depression revisited: a glimmer of hope. J Pers Med.

[5] Kas MJ, Do KQ, Sand MS (2026). Biomarker innovations in precision psychiatry diagnostics and treatment strategies. Eur Neuropsychopharmacol.

[6] Zhou C, Zhang H, Qin Y (2018). A systematic review and meta-analysis of deep brain stimulation in treatment-resistant depression. Prog Neuropsychopharmacol Biol Psychiatry.

[7] Kritzer MD, Peterchev AV, Camprodon JA (2023). Electroconvulsive therapy: mechanisms of action, clinical considerations, and future directions. Harv Rev Psychiatry.

[8] Ramos MR, Goerigk S, Aparecida da Silva V (2025). Accelerated theta-burst stimulation for treatment-resistant depression: a randomized clinical trial. JAMA Psychiatry.

[9] Levy MJ, Boulle F, Steinbusch HW, van den Hove DL, Kenis G, Lanfumey L (2018). Neurotrophic factors and neuroplasticity pathways in the pathophysiology and treatment of depression. Psychopharmacology (Berl).

[10] Duman RS, Deyama S, Fogaça MV (2021). Role of BDNF in the pathophysiology and treatment of depression: Activity-dependent effects distinguish rapid-acting antidepressants. Eur J Neurosci.

[11] Arosio B, Guerini FR, Voshaar RC, Aprahamian I (2021). Blood brain-derived neurotrophic factor (BDNF) and major depression: do we have a translational perspective?. Front Behav Neurosci.

[12] Sanjari Moghaddan H, Akhondzadeh S (2023). The clinician scientist training program in Iran: catalyzing clinical science advancements. Avicenna J Med Biotechnol.

[13] Loef D, Tendolkar I, van Eijndhoven PF (2023). Electroconvulsive therapy is associated with increased immunoreactivity of neuroplasticity markers in the hippocampus of depressed patients. Transl Psychiatry.

[14] Schmidt HD, Duman RS (2010). Peripheral BDNF produces antidepressant-like effects in cellular and behavioral models. Neuropsychopharmacology.

[15] Gejl AK, Enevold C, Bugge A, Andersen MS, Nielsen CH, Andersen LB (2019). Associations between serum and plasma brain-derived neurotrophic factor and influence of storage time and centrifugation strategy. Sci Rep.

[16] Fidalgo TM, Morales-Quezada JL, Muzy GS (2014). Biological markers in noninvasive brain stimulation trials in major depressive disorder: a systematic review. J ECT.

[17] Meshkat S, Alnefeesi Y, Jawad MY (2022). Brain-derived neurotrophic factor (BDNF) as a biomarker of treatment response in patients with treatment-resistant depression (TRD): a systematic review & meta-analysis. Psychiatry Res.

[18] Madsen CA, Navarro ML, Elfving B, Kessing LV, Castrén E, Mikkelsen JD, Knudsen GM (2024). The effect of antidepressant treatment on blood BDNF levels in depressed patients: a review and methodological recommendations for assessment of BDNF in blood. Eur Neuropsychopharmacol.

[19] Zhou D, Li X, Wei S (2024). Transcranial direct current stimulation combined with repetitive transcranial magnetic stimulation for depression: a randomized clinical trial. JAMA Netw Open.

[20] Polacchini A, Metelli G, Francavilla R, Baj G, Florean M, Mascaretti LG, Tongiorgi E (2015). A method for reproducible measurements of serum BDNF: comparison of the performance of six commercial assays. Sci Rep.

[21] Brunoni AR, Machado-Vieira R, Zarate CA Jr (2014). BDNF plasma levels after antidepressant treatment with sertraline and transcranial direct current stimulation: results from a factorial, randomized, sham-controlled trial. Eur Neuropsychopharmacol.

[22] Murawska-Ciałowicz E, Wiatr M, Ciałowicz M (2021). BDNF impact on biological markers of depression-role of physical exercise and training. Int J Environ Res Public Health.

[23] (2022). Diagnostic and Statistical Manual of Mental Disorders, 5th ed., text rev.

[24] (2026). ICD-11: International Classification of Diseases 11th Revision. https://icd.who.int/en/.

[25] Nejadghaderi SA, Balibegloo M, Rezaei N (2024). The Cochrane risk of bias assessment tool 2 (RoB 2) versus the original RoB: a perspective on the pros and cons. Health Sci Rep.

[26] Stang A (2010). Critical evaluation of the Newcastle-Ottawa scale for the assessment of the quality of nonrandomized studies in meta-analyses. Eur J Epidemiol.

[27] Sanabria AJ, Rigau D, Rotaeche R, Selva A, Marzo-Castillejo M, Alonso-Coello P (2015). GRADE: Methodology for formulating and grading recommendations in clinical practice (Article in Spanish). Aten Primaria.

[28] Vanicek T, Kranz GS, Vyssoki B (2019). Acute and subsequent continuation electroconvulsive therapy elevates serum BDNF levels in patients with major depression. Brain Stimul.

[29] Xie XH, Xu SX, Yao L (2024). Altered in vivo early neurogenesis traits in patients with depression: evidence from neuron-derived extracellular vesicles and electroconvulsive therapy. Brain Stimul.

[30] Valiuliene G, Valiulis V, Dapsys K, Vitkeviciene A, Gerulskis G, Navakauskiene R, Germanavicius A (2021). Brain stimulation effects on serum BDNF, VEGF, and TNFα in treatment-resistant psychiatric disorders. Eur J Neurosci.

[31] Mindt S, Neumaier M, Hellweg R, Sartorius A, Kranaster L (2020). Brain-derived neurotrophic factor in the cerebrospinal fluid increases during electroconvulsive therapy in patients with depression: a preliminary report. J ECT.

[32] Permoda-Pachuta A, Malewska-Kasprzak M, Skibińska M, Rzepski K, Dmitrzak-Węglarz M (2023). Changes in adipokine, resitin, and BDNF concentrations in treatment-resistant depression after electroconvulsive therapy. Brain Sci.

[33] Sorri A, Järventausta K, Kampman O (2018). Effect of electroconvulsive therapy on brain-derived neurotrophic factor levels in patients with major depressive disorder. Brain Behav.

[34] Salehi I, Hosseini SM, Haghighi M (2016). Electroconvulsive therapy (ECT) and aerobic exercise training (AET) increased plasma BDNF and ameliorated depressive symptoms in patients suffering from major depressive disorder. J Psychiatr Res.

[35] Tong J, Zhang J, Jin Y (2021). Impact of repetitive transcranial magnetic stimulation (rTMS) on theory of mind and executive function in major depressive disorder and its correlation with brain-derived neurotrophic factor (BDNF): a randomized, double-blind, sham-controlled trial. Brain Sci.

[36] Bouckaert F, Dols A, Emsell L (2016). Relationship between hippocampal volume, serum BDNF, and depression severity following electroconvulsive therapy in late-life depression. Neuropsychopharmacology.

[37] Bilgen AE, Bozkurt Zincir S, Zincir S, Ozdemir B, Ak M, Aydemir E, Sener I (2014). Effects of electroconvulsive therapy on serum levels of brain-derived neurotrophic factor and nerve growth factor in treatment resistant major depression. Brain Res Bull.

[38] Gedge L, Beaudoin A, Lazowski L, du Toit R, Jokic R, Milev R (2012). Effects of electroconvulsive therapy and repetitive transcranial magnetic stimulation on serum brain-derived neurotrophic factor levels in patients with depression. Front Psychiatry.

[39] Pelosof R, Santos LA, Farhat LC, Gattaz WF, Talib L, Brunoni AR (2023). BDNF blood levels after electroconvulsive therapy in patients with mood disorders: an updated systematic review and meta-analysis. World J Biol Psychiatry.

[40] Rocha RB, Dondossola ER, Grande AJ (2016). Increased BDNF levels after electroconvulsive therapy in patients with major depressive disorder: a meta-analysis study. J Psychiatr Res.

[41] Albuquerque AN, Minervino CA, Costa RS, Alves PV, Araújo JV (2026). Electroconvulsive therapy, BDNF and major depressive disorder: a meta-analysis with biological subgroups and clinical assessment of outcomes. J Affect Disord.

[42] Noetel M, Sanders T, Gallardo-Gómez D (2024). Effect of exercise for depression: systematic review and network meta-analysis of randomised controlled trials. BMJ.

[43] Yuping Z, Tianbi L, Wentao S, Yun L, Guodong Z (2024). The optimal type and dose of exercise for elevating brain-derived neurotrophic factor levels in patients with depression: a systematic review with pairwise, network, and dose-response meta-analyses. Depress Anxiety.

[44] Brunoni AR, Baeken C, Machado-Vieira R, Gattaz WF, Vanderhasselt MA (2015). BDNF blood levels after non-invasive brain stimulation interventions in major depressive disorder: a systematic review and meta-analysis. World J Biol Psychiatry.

[45] Wang Z, Zou Z, Xiao J (2023). Brain-derived neurotrophic factor blood levels after electroconvulsive therapy in patients with mental disorders: a systematic review and meta-analysis. Gen Hosp Psychiatry.

